# Predicting Hydroxychloroquine Clearance in Healthy and Diseased Populations Using a Physiologically Based Pharmacokinetic Approach

**DOI:** 10.3390/pharmaceutics15041250

**Published:** 2023-04-15

**Authors:** Faleh Alqahtani, Ali Mohammed Asiri, Ammara Zamir, Muhammad Fawad Rasool, Amer S. Alali, Sary Alsanea, Ismail A. Walbi

**Affiliations:** 1 Department of Pharmacology and Toxicology, College of Pharmacy, King Saud University, Riyadh 11451, Saudi Arabia; 2 Department of Pharmacy Practice, Faculty of Pharmacy, Bahauddin Zakariya University, Multan 60800, Pakistan; 3 Department of Pharmaceutics, College of Pharmacy, Prince Sattam Bin Abdulaziz University, Al-Kharj 11942, Saudi Arabia; 4 Department of Clinical Pharmacy, College of Pharmacy, Najran University, Najran 64462, Saudi Arabia

**Keywords:** hydroxychloroquine, physiologically based pharmacokinetics, chronic kidney disease, liver cirrhosis

## Abstract

Hydroxychloroquine (HCQ), a congener of chloroquine, is widely used in prophylaxis and the treatment of malaria, and also as a cure for rheumatoid arthritis, systemic lupus erythematosus, and various other diseases. Physiologically based pharmacokinetic modeling (PBPK) has attracted great interest in the past few years in predicting drug pharmacokinetics (PK). This study focuses on predicting the PK of HCQ in the healthy population and extrapolating it to the diseased populations, i.e., liver cirrhosis and chronic kidney disease (CKD), utilizing a systematically built whole-body PBPK model. The time vs. concentration profiles and drug-related parameters were obtained from the literature after a laborious search and in turn were integrated into PK-Sim software for designing healthy intravenous, oral, and diseased models. The model’s evaluation was performed using observed-to-predicted ratios (Robs/Rpre) and visual predictive checks within a 2-fold error range. The healthy model was then extrapolated to liver cirrhosis and CKD populations after incorporating various disease-specific pathophysiological changes. Box–whisker plots showed an increase in AUC_0-t_ in liver cirrhosis, whereas a decrease in AUC_0-t_ was seen in the CKD population. These model predictions may assist clinicians in adjusting the administered HCQ doses in patients with different degrees of hepatic and renal impairment.

## 1. Introduction

Hydroxychloroquine (HCQ) was synthesized in 1946 as an analog of chloroquine by adding a hydroxyl group at the carbon number 7, and it exhibits fewer side effects but the same efficacy [1]. It is a notable drug recommended for the treatment and prophylaxis of different species of malaria disease [2]. Furthermore, it has demonstrated its efficacy against various disorders such as rheumatoid arthritis, systemic lupus erythematosus (SLE), discoid lupus erythematosus (DLE), palindromic rheumatism, eosinophilic fasciitis, dermatomyositis, Sjogren’s syndrome, porphyria cutanea tarda, polymorphous light eruption, granuloma annulare, lichen plannus, and lupus panniculitis [3]. In addition, HCQ was approved for use in coronavirus disease 2019 (COVID-19) prophylaxis and treatment [4]. HCQ has multiple mechanisms of action in various diseases [3,5], and it is commercially available in an oral dosage form as a 200 mg HCQ sulfate tablet equivalent to 155 mg base [6].

HCQ is a 4-aminoquinolone and belongs to the biopharmaceutical classification system (BCS) class I [7] and thus is readily absorbed after oral administration [8]. The complexity of HCQ pharmacokinetics (PK) comes from its large volume of distribution due to extensive uptake into tissues, leading to a long half-life of around 40–50 days [9]. HCQ is bound to α-1 acid glycoprotein with an unbound fraction (f_u_) of 0.48 [10]. It is mainly metabolized in the liver by de-alkylation through cytochrome P450 enzymes (CYP3A4, CYP2C8, CYP2D6) to its metabolites [11]. HCQ has a renal plasma clearance of 12.7 L/h, and the metabolites are mainly excreted renally [6,12]. Although HCQ is well tolerated and considered a less toxic derivative of chloroquine [13], it has reported some serious side effects such as cardiomyopathy [14] and retinopathy [15].

In 1937, Teorell initiated the physiologically based pharmacokinetic (PBPK) modeling approach for the first time, and its utilization has grown over the past years after its effectiveness and application were proven in different phases of drug development and improvement in terms of research. Recently, it has gained great interest due to its involvement in regulatory submissions, and apparently, it has raised the total number of published papers in this field [16]. PBPK modeling is a powerful computational technique that utilizes complex mathematical calculations for predicting drug response in virtual populations. It is a predictive tool for the PK behavior of drugs that integrates the different processes relating to drug kinetics, including absorption, distribution, metabolism, and elimination (ADME) along with the physicochemical properties of the drug compound [17].

PBPK modeling is one of the best tools used to describe the pathophysiological changes in patients with hepatic or renal abnormalities based on the disease severity to design a specialized dosing regimen. As HCQ is intended for treating different medical conditions, it is used by many populations, including patients with special considerations such as renal impairment and liver cirrhosis. Therefore, it is of clinical significance to observe the influence of liver and kidney functionality on HCQ PK to design an optimal dose regimen with maximum activity and minimum toxicity. Understanding the chemical structure of the compounds plays a significant role in predicting the PK and pharmacodynamic properties of the molecule [18]. Previously developed models have incorporated different reported pathophysiological changes occurring in the liver [19,20] and renal diseases [21], such as organ blood flows, glomerular filtration rate, liver organ volume, hematocrit, and plasma protein (in the case of liver cirrhosis) [22,23] and in gastric emptying time, small intestinal transit time, hematocrit, and glomerular filtration rate (in case of CKD) [24,25], which will be extrapolated in our model as well.

The PBPK models for HCQ have already been recently developed, in which one was focused on dose optimization of HCQ for specific populations in COVID-19 patients using concomitant medications [26], a second concentrated on determining the in vitro anti-viral activity of HCQ and its optimization of dose in SARS-CoV-2 [27], and a third one emphasized the modulation of the process of autophagy in cancer patients [28]. In short, the models developed until now have been explicitly related to COVID-19, SARS-CoV-2, and cancer patients. The novelty of the study is that, to date, no model for HCQ has been developed that is used to predict its exposure in patients of liver cirrhosis and CKD populations. Therefore, the present study was conducted to develop and evaluate the HCQ PBPK model in healthy adults after intravenous (IV) and oral administration, with the aim to extrapolate this model to CKD and liver cirrhosis populations after the incorporation of relevant disease-specific changes.

## 2. Materials and Methods

### 2.1. Observed Pharmacokinetic Data

A literature survey using online resources was conducted for screening clinical studies having an explicit systemic concentration profile of HCQ after IV and oral administrations. The selection criteria for the study are based on the existence of relevant information required for the simulations, which include demographic data (age, gender, and weight) of the volunteers enrolled in the study, female proportion, the dose given, and administration protocol (IV or oral) when available. Due to the wide range in the blood-to-plasma ratio of HCQ, Tett et al. [9] recommend using blood data over plasma measurements; therefore, the available PK data were from a whole blood measurement. By the end of the survey, 12 drug disposition profiles were obtained from 8 publications, of which 3 were obtained after IV administration, and the remaining 5 after oral applications, as shown in Table 1. The observed concentration–time curves from published papers were scanned using Get Data Graph Digitizer version 2.26 to convert these graphs into points for further use in model development and evaluation. For PBPK model development, one-third of the studies (1 IV and 2 oral) were utilized, whereas the rest of the two-thirds (2 IV and 3 oral) were used in the verification of the model. All of the studies were employed in the final evaluation of the model. Unfortunately, there are no available clinical data on the use of HCQ concerning hepatic and renal impairments.

### 2.2. Modeling Software and Design

#### Model Designing

**Figure 1:**
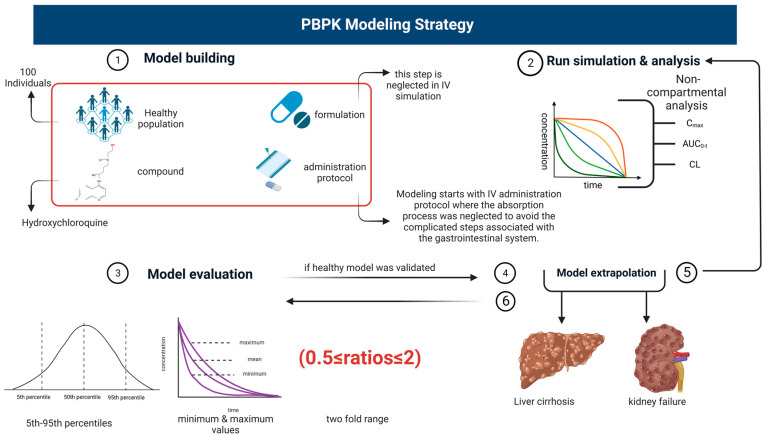
Schematic workflow diagram for the development of hydroxychloroquine PBPK model. The figure was created with BioRender.com with agreement number VR24U6YSZI.

An organized workflow was adopted to build up a PBPK model beginning with extensive online research using Google Scholar and PubMed to collect the reported values pertaining to drug-related parameters (MW, lipophilicity log P, fraction unbound fu, B/P ratio, drug solubility) that may have an impact on systemic drug exposure. After that, the respective parameters were incorporated into the PK-Sim^®^ simulator together with the simulation’s relevant anatomical and physiological data, based on the presence of systemic concentration-vs.-time profiles of HCQ in the published literature. Each profile has a self-governing simulation based on the characteristics (origin, age, weight, female %) found in the published data. The previously reported systematic model-building approach was used in developing and evaluating the HCQ PBPK model [21,33,34,35]. Modeling was initiated by creating 100 virtual adults after IV administration protocol, where the absorption process was neglected to avoid the complicated steps associated with the gastrointestinal system. After the simulation ends, the simulator generates a time-versus-concentration profile and non-compartmental analysis (NCA) data that can be used for model evaluation. When the IV model was effectively developed and evaluated, oral PK profiles were cast into place by considering the compound’s absorption. After all developed simulations had been assessed in healthy adults, the model was extrapolated to liver cirrhosis and CKD by incorporating the respective pathophysiological changes. The schematic diagram for model designing can be seen in Figure 1.

### 2.3. Model Parametrization

As HCQ has a complex PK profile, an expanded literature review was conducted to find the drug-related parameters: physiochemical properties, biological processes, and other required parameters to build a PBPK model. The values of different parameters can be seen in Table 2.

### 2.4. Software

In the current study, PK-Sim^®^ (version 11) (Bayer Technology Services, Computational Solutions, 51368 Leverkusen, Germany) was used to build a physiologically based pharmacokinetic model for HCQ in healthy and diseased populations.

#### 2.4.1. Absorption

HCQ is a basic drug given as sulfate salt absorbed in the upper portion of the intestine. The specific intestinal permeability was optimized manually to 1.06 × 10^−^^5^ cm/min based on visual predictive checks and a comparison of observed and predicted data. Moreover, Lint80 was used in the formulation method in which dissolution time and lag time were optimized manually to 300 min and 1 h, respectively.

#### 2.4.2. Distribution

The PK-Sim standard method was used for the estimation of both partition coefficient and cellular permeability.

#### 2.4.3. Metabolism

Due to the lack of intrinsic clearance (CL_int_) values of CYP enzymes (CYP3A4, CYP2C8, CYP2D6) responsible for HCQ metabolism, the calculation of the metabolic CYP enzyme values was performed using a well-stirred model [21]. In the beginning, the total hepatic intrinsic clearance (CL_int.H_) was calculated from the fraction unbound of the compound, blood-to-plasma ratio, and hepatic and renal clearance of HCQ. After the CL_int.H_, was calculated, the in vitro clearance of each enzyme was obtained by multiplying with the percentage contribution of the enzyme and by dividing by its abundance obtained from the software. The percentage contributions of CYP3A4, CYP2C8, and CYP2D6 were obtained from a previous study [26].

### 2.5. Model Predictions in Liver Cirrhosis and CKD Populations

The severity of liver cirrhosis is judged by the Child–Pugh (CP) scoring system. In this system, liver cirrhosis patients are categorized into CP-A, B, and C based on the scoring of 5 parameters, i.e., albumin, bilirubin, encephalopathy, prothrombin time, and ascites. Final scores of 5–6, 7–9, and 10–15 represent well-compensated disease (CP-A), compromised functional disease (CP-B), and decompensated disease (CP-C), respectively. The HCQ model was extrapolated in the liver cirrhosis population by incorporating the changes in values reported in the literature among different parameters such as organ blood flows, glomerular filtration rate, liver organ volume, hematocrit, and plasma protein [22,23].

Similarly, CKD is also categorized into moderate and severe renal failure based on the glomerular filtration rate. The HCQ model was further extrapolated to a chronic kidney disease (CKD) population by integrating the changes reported in previously published literature among different parameters such as gastric emptying time, small intestinal transit time, hematocrit, and glomerular filtration rate [24,25]. HCQ binds to α-1 acid glycoprotein, whose changes in values are not reported in the literature in the case of CKD. Moreover, for moderate and severe CKD, we have inputted glomerular filtration rate values as 45 and 20, respectively. Box-plots were made for comparison of AUC_0-t_ of the healthy population with all three classes of liver cirrhosis (CP-A, CP-B, and CP-C) as well as for moderate and severe CKD stages.

### 2.6. Evaluation of Developed PBPK Models

The model accuracy was evaluated on the criterion of whether observed data limits were within the 95% confidence interval of the predicted values. The predicted and observed PK parameters, e.g., the maximum concentration (C_max_), area under the curve (AUC), and plasma clearance (CL), were determined from NCA using the Microsoft Excel^®^ add-in PK-Solver program [36]. The predicted values are considered reasonable if the observed-to-predicted-data ratio was within a predefined 2-fold range. The model evaluation was performed using visual predictive checks (VPC), where the predicted and simulated plasma drug concentrations were overlaid along with 5th–95th percentiles and minimum and maximum values for the predictions. The observed and predicted PK parameters were compared to the calculated observed/predicted ratio (R_obs_/R_pre_). As used in previous PBPK-based studies, (R_obs_/R_pre_) parameters were considered acceptable if they were within a 2-fold error. After a comparison of ratios in a healthy model following IV and oral administration, one included healthy study [29] was used as a reference for comparison with the extrapolated diseased models (liver and kidney insufficiency). The equations used to calculate the intrinsic clearance and mean observed/predicted ratios were as follows:

(1) $${CL}_{int}=\frac{Q_{H}\times {CL}_{H}}{{fu}_{B}\times \left(Q_{H}-{CL}_{H}\right)}$$

(2) $$R=\frac{Observed\;value\;of\;PK\;parameter}{Predicted\;value\;of\;PK\;parameter}$$

## 3. Results

### 3.1. Intravenous and Oral Administration in the Healthy Population

**Figure 2:**
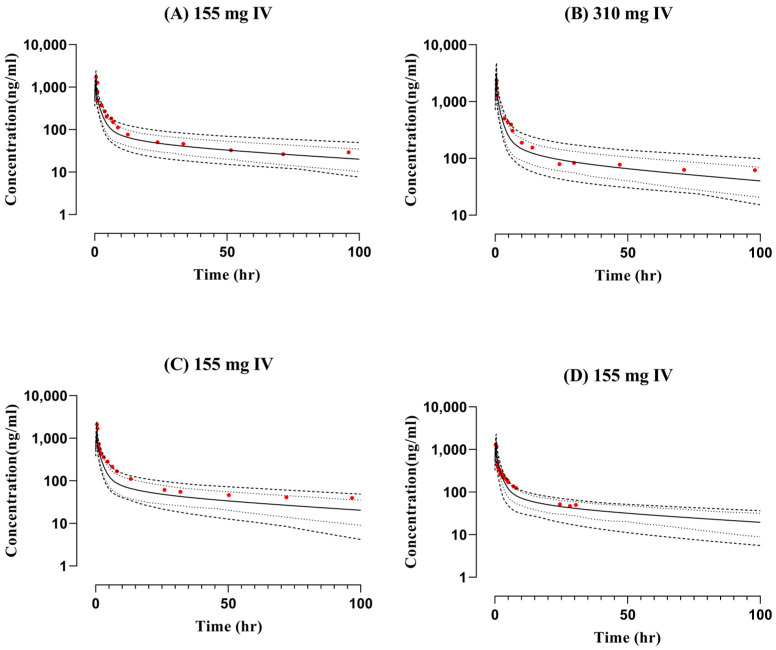
Comparison of observed and predicted blood-concentration–time profiles after intravenous administration of doses (**A**) 155 mg [9], (**B**) 310 mg [9], (**C**) 155 mg [6], (**D**) 155 mg [29]. Solid lines are presented as mean, dashed lines as minimum, maximum, dotted lines as 5th–95th percentile and red-filled circles as observed data.

**Figure 3:**
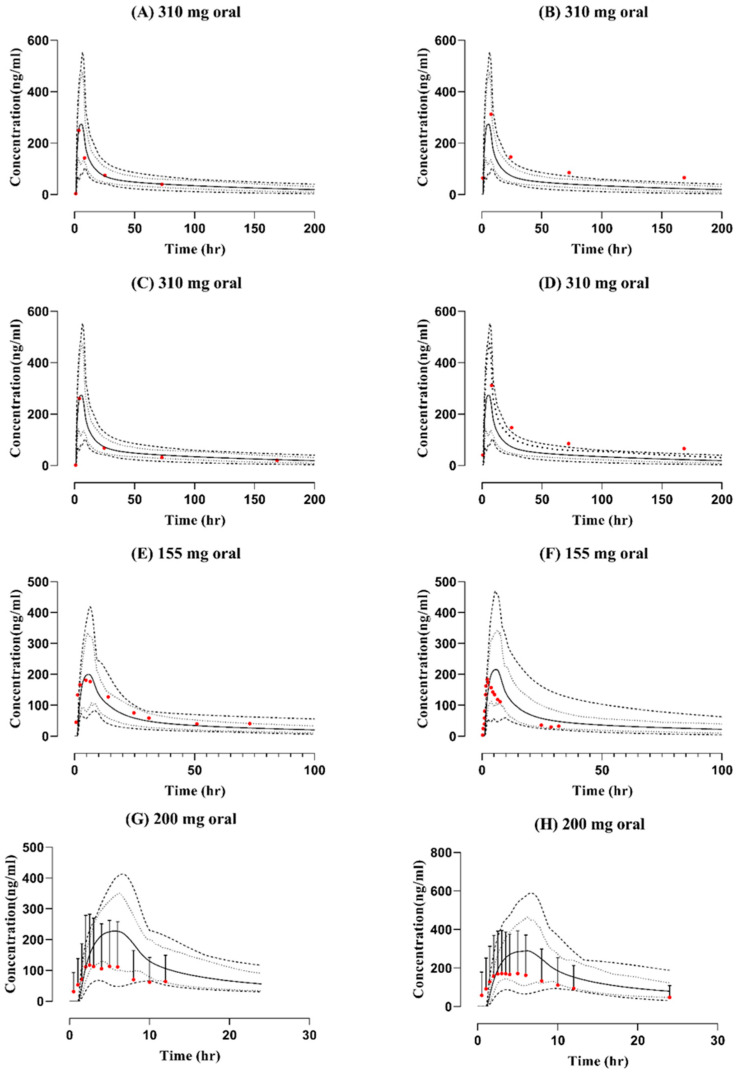
Observed and predicted blood concentration-time profiles comparison after oral administration of dose (**A**) 310 mg [30], (**B**) 310 mg [30], (**C**) 310 mg [30], (**D**) 310 mg [30], (**E**) 155 mg [6], (**F**) 155 mg [29], (**G**) 200 mg [31], (**H**) 200 mg [32]. Solid lines are presented as mean, dashed lines as minimum, maximum and dotted lines as 5th–95th percentile and red-filled circles as observed data.

The observed and simulated data from the blood-drug-concentration–time profiles after IV and oral application following the administration of doses (155 mg, 310 mg, and 200 mg) can be seen in Figure 2 and Figure 3. The visual predictive checks show that observed profiles fit with the simulated curves in comparison with the arithmetic mean, minimum, maximum, and 5th–95th percentiles. The model has successfully captured the data, which were further confirmed by the mean R_obs_/R_pre_ ratios for all pharmacokinetic parameters such as C_max_, AUC_0-t_, and CL. The mean R_obs_/R_pre_ ratios for AUC_0-t_ after IV administration ranged between 1.13 and 1.55, whereas after oral administration, it was between 0.54 and 1.94 and within the 2-fold error range. The ratios for C_max_ and CL can be seen in Table 3.

### 3.2. Extrapolation of the Model to Liver Cirrhosis and CKD

#### 3.2.1. Intravenous Administration

**Figure 4:**
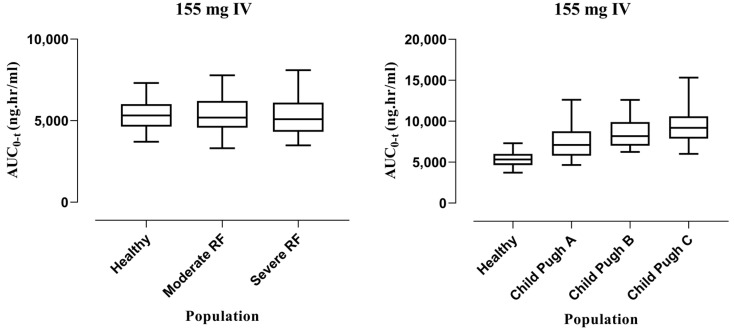
Box plots showing median with 5th–95th percentile after IV administration in liver cirrhosis and CKD.

After extrapolating the healthy model to liver cirrhosis and CKD population, model predictions were made following IV administration of 155 mg. In liver cirrhosis, the median (95% confidence interval (CI)) for AUC_0-t_ in healthy is 5317 (5142–5532), which increased to 7096 (6465–7528) in CP-A, 8176 (7772–8650) in CP-B, and 9204 (8645–9709) in CP-C, whereas for CKD, the value in the healthy population is 5317 (5142–5532) and decreased to 5187 (4904–5519) in moderate renal failure and 5088 (4665–5466) in severe renal failure. The box plots depicting these changes can be seen in Figure 4.

#### 3.2.2. Oral Administration

**Figure 5:**
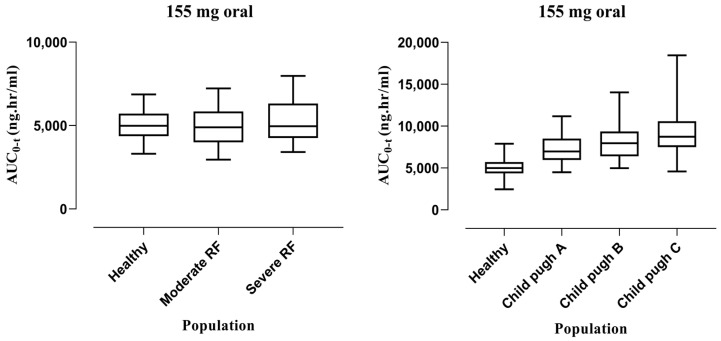
Box plots showing median with 5th–95th percentile after oral administration in liver cirrhosis and CKD.

After oral administration of 155 mg dose, the model was extrapolated from healthy to liver cirrhosis and CKD populations. The values of the median with 95% CI for AUC_0-t_ in the healthy population was 4989 (4812–5245), which increased to 6966 (6488–7600) in CP-A, 7961 (7171–8600) in CP-B, and 8735 (8449–9632) in CP-C, whereas for CKD the value in the healthy population was 4989 (4812–5245) and decreased to 4890 (4539–5235) in moderate renal failure and 4961 (4603–5342) in severe renal failure. The box plots showing these changes can be seen in Figure 5.

## 4. Discussion

This study used a systematic approach for building the HCQ PBPK model in the healthy and diseased populations (liver cirrhosis and CKD) after the administration of doses through IV and oral routes. In continuity with previously published models, the model evaluation was performed in the healthy population first, which was then extrapolated to the diseased population. The PK of HCQ was distinguished by three features: high distribution volume, long terminal half-life, and low plasma clearance. Noticeably, HCQ is metabolized principally in the liver into des-ethyl hydroxychloroquine and des-ethyl chloroquine via cytochrome P450 enzymes through CYP3A4, CYP2C8, and CYP2D6. These drug-metabolizing enzymes are altered in response to impaired organ function.

In the presented work, we sought to develop and validate a PBPK model for IV and oral HCQ model in healthy individuals through an in silico technique, depending on the available reported physiochemical properties of the compound and concentration profiles from studies in the literature. The results show that the observed and predicted values were consistent with each other, as evident from the mean AUC_0-t_ value of 1857.45 ng.hr/mL vs. 1568.5 ng.hr/mL after IV administration. Similarly, the mean observed AUC_0-t_ value of 223.45 ng.hr/mL was in line with that of the predicted AUC_0-t_ value of 239.76 ng.hr/mL following oral administration. In the visual presentation, the red dots (observed data) were not superimposed on the predicted line, but the results were comparable to the already developed published models on SARS-CoV2, COVID-19, and cancer patients [26,27,28]. Although the model results are within a 2-fold error range, their quality is compromised, possibly due to the huge variability in the reported PK of HCQ.

HCQ is used as long-term therapy for autoimmune diseases, especially rheumatoid arthritis and systemic lupus erythematosus. Patients in such cases could use this drug for over 10 years which shows the importance of its PK properties. The already published PBPK model on HCQ in COVID-19 patients has recommended that the dose needs to be reduced to meet its desired response, which in turn explains the importance of the development of the updated model and requires its extrapolation to other diseased and specific populations [26]. Jallouli et al. [37] analyzed the factors associated with drug concentration variability among SLE patients and found that renal dysfunction increased the systemic blood concentration of HCQ but was not significantly altered and was still in the therapeutic window. Moreover, two other studies also reported an increase in blood concentration of HCQ during renal impairment [38,39]. In the extrapolation of the model to CKD, disease-specific pathophysiological changes such as gastric emptying time, small intestinal transit time, hematocrit, and glomerular filtration rate were integrated [24,25]. The box plots have shown that AUC_0-t_ decreased by 2.44% and 4.30% in cases of moderate and severe renal failure following IV administration and about 1.98% and 0.56% following oral administration. This decrease in AUC_0-t_ may be due to a decrease in plasma protein binding and in turn an increase in metabolic clearance of drugs, thus suggesting the monitoring of doses in this case (moderate and severe renal impairment). The model predictions have shown no significant changes in HCQ exposure, and to confirm these findings clinical PK data for HCQ is required, which to date is not available in the published literature.

HCQ is a drug with a low hepatic extraction ratio, which showed that if blood flow towards the liver is reduced in the case of cirrhosis, it would not cause any direct increase in the exposure to the drug [6], but to guide clinicians regarding dosing, consideration should be given to reducing the dose irrespective of this plasma concentration effect, as HCQ has proven to be accumulated in the liver in animal studies [40]. As the liver and kidney are the main sites involved in drug metabolism and excretion of HCQ, the pathophysiological changes of the organ dysfunction were incorporated based on the impairment severity in liver cirrhosis. The changes in values are taken from the reported literature among different parameters that include organ blood flows, glomerular filtration rate, liver organ volume, hematocrit, and plasma protein in liver cirrhosis [22,23]. The AUC_0-t_ was found to be increased by 33.5%, 53.77%, and 73.10% in CP-A, CP-B, and CP-C, respectively, after IV administration, whereas in the case of oral administration, the increase was more pronounced at 39.62%, 59.57%, and 75.08%, respectively. This may be due to changes in hepatic blood flow followed by increased bioavailability of hydroxychloroquine, suggesting that dosage adjustment may be required in hepatic impairment.

### Limitations

Due to the huge variability in the reported PK parameters, the visual predictive checks showed that the predictions were within the 5th–95th percentile but were not in close agreement with the reported data, which may reduce the reliability of the results. The HCQ clinical PK data are limited to the healthy population, and no data are available in CKD and liver cirrhosis populations; therefore, the predicted data in these disease populations could not be verified. Moreover, it is known that plasma protein binding is altered in CKD, but there is no reported value for changes in α-1 acid glycoprotein in this disease. Therefore, no change in α-1 acid glycoprotein concentration was incorporated into the developed CKD model. In addition, due to a lack of clear information on CYP enzyme CL_int_ values in the literature, a well-stirred liver model was used to back-calculate the CL_int_ of metabolic enzymes. The plasma-concentration–time profiles of HCQ were scanned to extract PK parameters that were in line with published data. Still, there is a possibility of some minor differences that cannot be avoided.

## 5. Conclusions

The developed HCQ PBPK model has successfully described the PK in healthy and diseased populations (liver cirrhosis and CKD). The extrapolation of the model to liver cirrhosis and CKD populations with different stages of severity resulted in significant changes in liver cirrhosis but not in CKD; therefore, the adjustment of doses for HCQ may be required in hepatically impaired patients.

## Figures and Tables

**Table 1:** List of clinical PK data of hydroxychloroquine obtained from published studies utilized in PBPK model development.

Sr. No.	Study ID	Dose (mg)	Infusion Time	N	Female (*n*) %	Mean Age (years)	Mean Weight (kg)	Population
**Hydroxychloroquine administered by intravenous infusion**								
1-	Tett et al. (1988) [9]	155	30 min	5	60	22.6 (range: 19–27	63.5 (range: 55–68)	Healthy
		310						
2-	Tett et al. (1989) [6]	155	30 min	5	60	22.6 (range: 19–27	63.5 (range: 55–68)	Healthy
3-	Tett et al. (1992) [29]	155	30 min	9	66	24.4 (range: 20–28)	71.4 (range: 51–92.5)	Healthy
**Hydroxychloroquine administered orally**								
1-	Sharyon B. Williams et al. (1988) [30]	310	NA	2	N/R	NA	84	Healthy
							88	
2-	Tett et al. (1989) [6]	155	NA	5	60	22.6 (range: 19–27	63.5 (range: 55–68)	Healthy
3-	Tett et al. (1992) [29]	155	NA	9	66	24.4 (range: 20–28)	71.4 (range: 51–92.5)	Healthy
4-	J. Ducharme et al. (1995) [31]	200	NA	24	0	20–36	72–88	Healthy
5-	Liu. Y.-M et al. (2012) [32]	200	NA	Test: 27	0	20.1–30.0	51.0–74.8	Healthy
				Reference: [27]		20.1–27.9	51.9–71.5	

**Table 2:** Drug-related parameters that will be used in PK-sim software for the development of the PBPK model for Hydroxychloroquine.

Parameter	Input Value	Reference
Physicochemical properties		
Molecular weight (g/mol)	335.87	[6]
Lipophilicity (Log P)	2.4	Optimized value
Dissociation constant (pKa)	8.27, 9.67	[8]
ADME properties		
Blood to plasma ratio (B/P)	7.2	[26]
Fraction unbound plasma (f_u_)	0.48	[12]
Plasma protein binding partner	alpha-1 acid glycoprotein	[12]
Solubility of hydroxychloroquine sulfate (phosphate-buffered saline)	5 mg/mL (at pH 7.2)	[30]
Intestinal permeability	1.06 × 10^−5^ cm/min	Optimized value
In vitro clearance (CYP2C8) (ul/min/pmol)	0.0078	Back calculated by using a well-stirred liver model
In vitro clearance (CYP2D6) (ul/min/pmol)	0.025	
Intrinsic clearance (CYP3A4) (ul/min/pmol)	0.002	
Partition coefficients	PK-Sim standard	
Cellular permeability	PK-Sim standard	
Renal plasma clearance (CL_R_)	12.7 L/h	[26]

**Table 3:** Mean R_obs_/R_pre_ ratios of various pharmacokinetic parameters after intravenous and oral administration of hydroxychloroquine in the healthy population.

	C_max_ (ng/mL)			AUC_0-t_ (ng/mL·hr)			CL(L/h)		
Study	Observed	Predicted	R Ratio	Observed	Predicted	R Ratio	Observed	Predicted	R Ratio
IV									
1988	1716.74	1457.96	1.17	6587.57	5405.63	1.21	16.9	20.6	0.82
1988 310	2321.71	2017.4	1.15	12,607.25	11,122.39	1.13	12	20.36	0.6
1989	2105.9	1517.82	1.38	8649.54	5553.82	1.55	10.2	20.27	0.51
1992	1287.03	1280.82	1	3984.54	3364.52	1.18	31.05	26.85	1.15
Oral									
1988 vol * 1 wk * 1	249.18	251.96	0.98	5812.48	6566.34	0.88	38.94	37.98	1.02
1988 vol 1 wk 4	311.932	236.80	1.31	17,952.57	9209.71	1.94	10.06	25.59	0.5
1988 vol 2 wk 1	261.05	264.16	0.98	8237.36	9667.94	0.85	28.78	24.59	1.17
1988 vol 2 wk 4	311.932	236.80	1.31	17,938.45	9210.98	1.94	10.12	25.51	0.5
1989	180.77	197.96	0.91	5470.81	4596.05	1.19	21.56	24.05	0.89
1992	183.5	214.80	0.85	2432.33	3680.55	0.66	52.32	28.29	1.84
1995	117.427	226.99	0.51	988.14	1799.07	0.54	110.03	76.76	1.43
2012	171.833	288.66	0.59	2446.55	3771.88	0.64	61.33	38.94	1.57

* vol: volunteer, * wk: week.

## Data Availability

All the data generated during the research are reported in the manuscript.
